# Oral Lesions as the Primary Manifestations of Behçet’s Disease: The Importance of Interdisciplinary Diagnostics—A Case Report

**DOI:** 10.3390/biomedicines11071882

**Published:** 2023-07-03

**Authors:** Alvaro Cavalheiro Soares, Fabio Ramoa Pires, Nara Regina de Oliveira Quintanilha, Lilian Rocha Santos, Thaylla Nunez Amin Dick, Arkadiusz Dziedzic, Bruna Lavinas Sayed Picciani

**Affiliations:** 1 Postgraduate Program in Dentistry, Nova Friburgo Health Institute, Fluminense Federal University, Nova Friburgo 28625-650, Brazil; 2 Department of Oral Pathology, School of Dentistry, Rio de Janeiro State University, Rio de Janeiro 20551-030, Brazil; 3 Independent Researcher, Niteroj 24230-101, Brazil; 4 Postgraduate Program in Pathology, School of Medicine, Fluminense Federal University, Niterói 24230-340, Brazil; 5 Department of Conservative Dentistry with Endodontics, Medical University of Silesia, 40-055 Katowice, Poland

**Keywords:** Behçet’s syndrome, Behçet’s disease, oral lesions, ulcers, vasculitis, inflammation, oral manifestations, diagnosis, genotyping, case report

## Abstract

Background: Behçet’s disease (BD) is a rare chronic auto-inflammatory systemic disease with non-specific oral manifestations, categorised as generalised variable vessel vasculitis that requires an interdisciplinary approach to diagnose due to its phenotypic heterogeneity. Whilst the oral lesions that reoccur in BD underpin the complex diagnostic process, the crucial role of dental professionals is highlighted in a case report summarised herein. We present a case of a 47-year-old male referred to the Oral Medicine Department by a rheumatologist after previous hospitalization for thrombosis of the iliac vein and inferior vena cava. He had elevated inflammatory C-reactive protein biomarker and an increased erythrocyte sedimentation rate. Recurrent episodes of folliculitis, oral and genital ulcers were reported. Clinical examination revealed multiple ulcerations in the oral mucosa. The complementary, histopathological analysis performed to rule out other disorders, based on excisional biopsy, showed non-keratinised stratified squamous epithelium with areas of exocytosis and ulceration. The connective tissue presented an intense mixed inflammatory infiltrate, congested blood vessels, haemorrhage, vasculitis, and HLA-B genotyping identified the expression of HLA-B15, further supporting the BD diagnosis. Treatment was initiated with colchicine, prednisolone, and weekly subcutaneous administration of methotrexate and resulted in the complete remission of oral lesions and no recurrence of other manifestations. Conclusions: This BD case report emphasizes the importance of a multidisciplinary approach in diagnosing BD, including the use of histopathological assessment and genetic profiling. It highlights the significance of thorough intraoral assessment and referral to a multidisciplinary team for diagnosis. The oral manifestations of BD as the primary symptoms often indicate underlying major systemic pathologies. The authors stress the need for a structured diagnostic algorithm to facilitate timely and effective management of BD.

## 1. Introduction

Behçet’s syndrome, also known as Behçet’s disease (BD) or oculo-orogenital syndrome, is a chronic auto-inflammatory disorder that affects multiple systems and is classified as a generalised vasculitis [1,2]. The trio of recurrent symptoms, aphthous stomatitis, genital ulcers, and iritis noted among native Mediterranean persons, were first described by the Turkish dermatologist, Hulusi Behçet in 1937 [3]. The prevalence of BD is considerably higher in regions near the Mediterranean Sea along the ancient Silk Route, including the Iberian Peninsula, Iran, Iraq, and Syria from the middle to the far east [1,2,4,5]. From an epidemiological perspective, BD has a global distribution. Although its exact pathogenesis is a medical conundrum that has yet to be fully understood, studies suggest that it involves a complex interaction between environmental factors in genetically susceptible individuals leading to the autoimmune reactivity [1,4,5,6].

Most recently, BD has been classified as part of a broader concept of major histocompatibility complex (MHC-I-opathy) pathologies. This classification considers the immunopathogenic basis shared by BD and clinically distinct spondyloarthropathies, such as reactive arthritis, psoriatic arthritis, ankylosing arthritis, and arthritis related to inflammatory bowel disease [7,8]. It is hypothesised that immunological alteration, an abnormal adaptive and innate immune response triggered by specific microorganisms could play a role in the initiating induction of BD [9,10,11]. Additionally, the presence of a permissive genetic background, particularly the HLA-B51 (Human Leucocyte Antigen, HLA) genetic profile, is considered a significant genetic co-factor associated with the onset and progression of BD [12,13,14]. However, the association between HLA-B51 and BD is not well-established outside the regions of the Silk Route. Reportedly, HLA-B51 is associated with severe disease manifestations such as progressive central nervous disease and posterior uveitis [8,12,14].

Amid BD’s wide spectrum of phenotypic heterogeneity, rarity, and complex immunopathogenesis, its diagnostic process can be hampered. Clinical challenges arise from the non-homogeneous manifestations and varying diagnostic guidelines. The medical dilemmas that exist for BD include geographical occurrence, pathognomonic diagnostic criteria, potential biomarkers, pathogenesis, and the therapeutic strategies. Considerable disparities among specialists exists regarding the concept clinical features and diagnosis [15].

BD is characterized as a chronic systemic inflammatory vasculitis primarily manifested by recurrent non-pathognomonic oral and genital ulcers, as well as skin lesions in the form of erythema nodosum, pseudofolliculitis, ocular lesions (anterior or posterior uveitis) (Table 1) [1,2,3,4,5]. Histopathological features play a crucial role in the differential diagnosis of BD. Although less prevalent, BD can also affect the nervous and gastrointestinal systems [1,2,4,5,6,7,8,16,17]. Recurrent oral ulcers, which are non-specific mucocutaneous manifestations, significantly impact quality of life and normal functioning of individuals [18]. What is more, various systemic manifestations, such as deep vein thrombosis (DVT), aneurysm development, and the nervous system involvement, although rarer, can lead to severe impairment of vital functions and even death [2,4,5,6,7,19]. The exact prothrombotic pathogenesis in BD remains unclear, but a vascular defect is deemed a primary cause [20,21], associated with impaired fibrinolysis [22], elevated procoagulant markers [23,24], and defective platelet function [25]. Fernandez-Bello et al. [26] indicated a likelihood of endothelium damage/activation as a contributing factor to the hypercoagulation state and disease activity.

According to a study conducted by Saadoun et al. [27], 5% of patients with BD died 7.7 years (median, 95% male) after follow-up due to major vessel diseases (arterial aneurysm, Budd–Chiari Syndrome), cancer, malignant hemopathy, or central nervous system disorders. The mean age of death was 34.8; hence, early recognition and the timely management of BD are crucial for improving outcome. This includes the essential role of dentists and oral medicine specialists who provide essential information about BD oral manifestations and their management [28].

**Table 1:** Common characteristics of histopathological features in Behçet’s Syndrome (based on [1,4,5,29]).

Primary Findings	Characteristic Features
Mucocutaneous lesions	Recurrent oral ulcerations. Recurrent genital ulcerations (aphthous, ‘herpetiform’). Cutaneous lesions: papulopustular lesions, nodosum-like lesions, pseudofolliculitis, erythema nodosum, acneiform nodules. Ocular lesions: uveitis, retinal vasculitis, cells in vitreum. Thrombophlebitis. Positive pathergy test.
Histopathological features and findings	Inflammatory infiltration of lymphocytes, macrophages, neutrophils at the base of ulcer, sometimes penetrating epidermidis. Infiltrate in perivascular regions. Neutrophil/lymphocyte exocytosis. Ulceration of epithelium. Inflammatory infiltrate with connective tissue. Vasculitis: neutrophilic vasculitis, lymphocytic vasculitis. Necrobiosis. Basal keratinocyte vacuolization. Oedema in dermis. Areas of microhemorrhages, congested vessels. Granular IgM and C3 deposits in dermoepidermal junction and in perivascular regions. IgM deposits at the vessel’s walls. Perivascular infiltrate of mononuclear cells. Presence of mast cells. Intraepidermal pustules. Spongiosis.

Considering the epidemiological characteristics, BD is typically diagnosed in young adults between the ages 30 and 40, with similar gender distribution. The appearance of oral and genital ulcerations is the most common symptom that leads individuals to seek medical attention [1,2,4,5]. The prevalence of BD varies globally, and it is estimated at 0.58 per 100,000 population in the Middle East, with a high prevalence in Turkey up to 420/1,000,000 population [30]. BD is rare in children and individuals older than 50 years. Scarce epidemiological data shows the prevalence of BD in Europe and the US between 0.12 and 7.5 per 100,000 population, with the most recent overall incidence estimate of 0.38 per 100,000 population and 5.2 per 100,000 prevalence in the US [5,30,31,32]. Regional differences in clinical characteristics have been reported, with less common ocular lesions and familial occurrence in the Western population [30]. Oral ulceration is often the initial sign of BD in about 80 to 95% of patients [5,33]. The unfavourable prognosis, indicating multisystemic involvement is attributed to young male patients due to severe vessels-related complications in complex cases [1,5,6,17,34].

BD typically manifests and follows a cycle of exacerbations (flare ups) and remission, with varying severity, duration, and the organs/systems involvement between episodes [4,5]. BD can pose serious and potentially life-threatening complications such as strokes and vision loss [1,4,5,6]. Various aspects of BD remain controversial or subject to debate, including the BD specific diagnostic criteria, pathogenesis, systemic impact, classification, and therapeutic recommendations. Diagnostic dilemmas occur while dealing with non-homogeneous clinical manifestations with various locations in people with systemic vasculitis with multiorgan involvement [35].

Since there is currently no specific test, the diagnosis of BD is based on the analysis of the specific patterns of clinical symptoms and their recurrent nature. Other potential causes of symptoms must be ruled out before a diagnosis of BD is made to facilitate an appropriate therapy [4,5,6,7]. The diagnostic strategy and clinical guidance of BD vary, leading to inconsistencies in the recommended criteria and not well-established level of certainty for BD diagnosis. The existing guidelines include the International Study Group (ISG) diagnostic guidelines (1990, Table 2) [36], the International Criteria for Behçet’s Disease (ICBD) (2013) [37], the National Health Service, England Diagnosis–Diagnosing Behçet’s Disease (2013) [38], and the revised version the International Team for the Revision of the International Criteria for Behçet’s Disease (ITR-ICBD, 2014) [39]. Multi-disciplinary cooperation unifying these guidelines is required to facilitate the optimal diagnostic pathway for BD. For practical purposes in general practice, simplified criteria of National Health Service England Diagnosis–Diagnosing Behçet’s Disease (2019) are applied, which consider the presence of primary BD symptoms—genital and mouth ulcers, red, painful eyes and blurred vision, acne-like spots, headaches, and painful, stiff, swollen joints [38].

Specialist tests can be used to support BD diagnosis and exclude other causes. These tests may include blood tests, urine tests, a skin biopsy, a pathergy test, radiographs, a computed tomography (CT) scan, and magnetic resonance imaging (MRI) [1,5,6,16,17,38,40]. While no specific laboratory or medical imaging test is currently recommended for BD, certain laboratory tests can help exclude a range of other systemic diseases. These tests include antineutrophil cytoplasmic antibody (ANCA), complete blood count, urinalysis, C-reactive protein (CRP) test and an erythrocyte sedimentation rate (ESR) test [1,38].

As BD may have a severe impact on an individual’s general health and well-being, causing long-term consequences, this case report highlights the importance of interdisciplinary collaboration in the diagnosis of BD. It emphasizes the involvement of oral medicine specialists, pathology teams, as well as genetic profiling in the diagnostic process. By presenting a detailed case, the report aims to provide insight into the manifestations of BD and underscore the need for a comprehensive diagnostic approach. Additionally, the report discusses the challenges and prospects associated with the diagnostic process of BD and it recognizes the lack of standardized diagnostic criteria for BD. The range of existing diagnostic guidelines is presented, highlighting variations among them. Here, this report suggests the need for multidisciplinary and unified approach to facilitate an effective diagnostic pathway for BD.

The preparation and organization of the case report follow the Clinical Case Reporting Guideline (CARE), ensuring a systematic and transparent presentation of the information. By adhering to this guideline, the report provides a comprehensive and reliable account of the diagnostic dilemmas and strategies related to BD.

## 2. Patient Information, Oral Manifestations, Diagnosis, and Therapy

A 47-year-old male patient was referred by a rheumatologist to the Oral Medicine Department complaining of recurrent episodes of painful oral lesions that have been present since his youth, affecting his daily functioning. The patient also had a history of previous hospitalisation due to extensive thrombosis of the iliac vein and inferior vena cava, as well as recurring pseudofolliculitis and genital ulcers. Laboratory tests, particularly a full blood count, showed persistently high levels of CRP and ESR.

### 2.1. Clinical Findings

**Figure 1:**
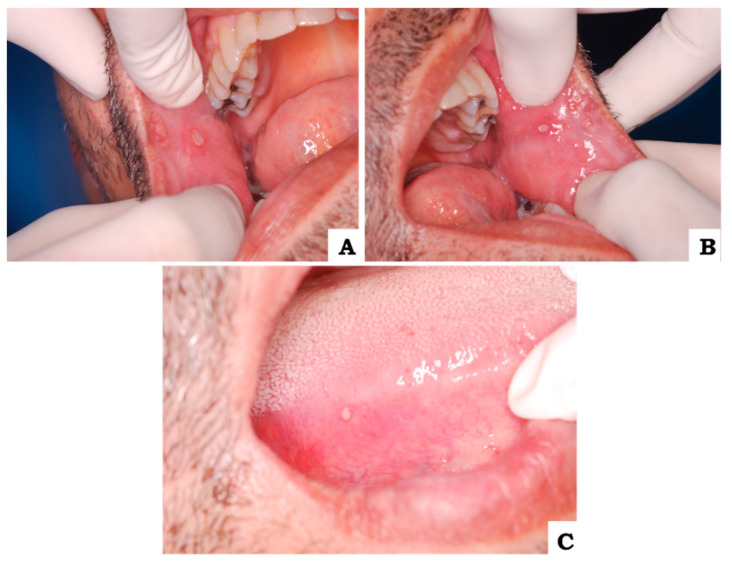
(**A**) clinical photograph of ulcerated lesion in right buccal mucosa subjected to excisional biopsy. (**B**) clinical photograph of ulcerated lesion in left buccal mucosa. (**C**) clinical photograph of ulcerated lesion in the right lateral border of the tongue.

During intraoral examination, multiple painful shallow ulcers were observed. The largest of which was approximately 0.7 cm in size and they were dispersed across the right buccal mucosa (Figure 1A), left buccal mucosa (Figure 1B), and the right lateral edge of the tongue (Figure 1C). The ulcers had an erythematous halo and a yellowish-white pseudomembrane, and they had been present for five days before the consultation. The patient did not report any other lesions on the body during the consultation, although he had experienced previous episodes of genital lesions. Given the clinical presentation, several conditions with similar clinical features were considered in the initial differential diagnosis. These included recurrent aphthous stomatitis, medication-induced oral lesions, inflammatory bowel disease (Crohn’s disease), Sweet syndrome, cyclic neutropenia, herpes infections, erythema multiforme, infection of human immunodeficiency virus (HIV), as well as various autoimmune diseases. Therefore, further investigations were necessary to establish a definite clinical diagnosis, rule out other underlying conditions, and confirm clinical diagnosis.

### 2.2. Additional Diagnostic Tests; Histopathological Assessment and HLA Genetic Profiling

**Figure 2:**
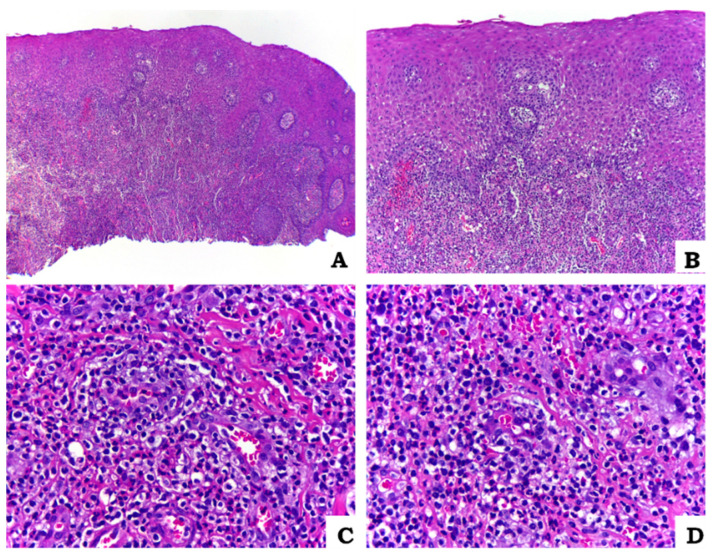
Histopathological examination of oral mucosa sample. Microscopic findings of the biopsy performed on the right buccal mucosa. Fragment of mucosa coated by non-keratinized stratified squamous epithelium with areas of exocytosis and ulceration ((**A**), HE 40×; (**B**), HE 100×). Further details of the underlying connective tissue showing intense mixed inflammatory infiltrate associated with haemorrhage, congested vessels ((**C**), HE 400×) and vasculitis areas ((**D**), HE 400×).

Despite the diagnosis of BD being established based on predominantly clinical manifestations, to further investigate the oral lesions, as a complementary test, an excisional biopsy of the right buccal mucosa lesion was performed for subsequent pathological examination and genotyping for HLA-B allele typing. This biopsy aimed to provide additional information and rule out other disorders with similar mucosal ulcerations, as well as confirm pathological features associated with generalized vasculitis. The histopathological examination revealed a fragment of mucosa covered by non-keratinised stratified squamous epithelium. Areas of exocytosis and ulceration were observed (Figure 2A,B). The underlying connective tissue was permeated by an intense mixed inflammatory infiltrate, with areas showing congested blood vessels, haemorrhage (Figure 2C) and vasculitis (Figure 2D). Interestingly, the HLA-B genotyping showed a negative result for HLA-B51 but a positive result for HLA-B15. This information adds to the diagnostic process and may help in further characterizing the patient’s genetic profile associated with BD.

No distinctive pathognomonic signs taken into consideration during the initial differential diagnosis process were observed. The patient did not report the use of medications that could be associated with the symptoms of other infectious pathologies, despite persistently elevated CRP and ESR parameters. Clinical symptoms and additional examinations did not suggest Crohn’s disease, Sweet syndrome, cyclic neutropenia, immune deficiency, as well as autoimmune disease. Based on a structured diagnostic process, primarily clinical characteristics supported by supplementary tests, the presence of vasculitis in the pathological examination and the positivity for HLA-B15 contributed to the diagnosis. The diagnostic clinical criteria recommended by both ISG and ICBD were applied [36,37]. The periodical occurrence of oral lesions provided the main evidence that initiated the process of differential diagnosis and detailed clinical investigation.

### 2.3. Therapeutic Intervention, Follow-Up and Outcome

Following consultation with a specialist team, systemic pharmacological therapy was initiated, and the patient started daily and continuous oral treatment with 0.5 mg colchicine and 20 mg prednisolone. Additionally, subcutaneous injections of 1 mL methotrexate 25 mg/mL were administered on a weekly basis. With this treatment regimen, a complete remission of the lesions was observed within approximately 60 days. Currently, the patient is under monthly follow-up and receiving specialist multidisciplinary care. During the last 8 months, there have been no recurrences of any manifestations of BD, indicating a positive outcome of the treatment.

## 3. Discussion

The comprehensive assessment of the patient’s clinical findings and the consideration of differential diagnoses highlight the complexity of diagnosing BD and the need for advanced investigations to differentiate it from other conditions with similar manifestations. Considering the epidemiology, in general, BD cases are rare in South America, including Brazil due to the geographical factors and because of Brazilian multi-ethnicity. Thus, the diagnosis of BD can be particularly challenging at a primary care level, since there is lack of familiarity with BD among health professionals, adding to the difficulty of recognizing the condition [33]. In addition, the early detection of BD might be further complicated by non-consistent clinical manifestations affecting multiple organs and tissues [41]. From a purely epidemiological point of view, the demographic profile of the male patient corroborates the findings of the literature that indicate that BD may manifest more severely in young men, especially regarding the vascular involvement and, exemplified in this case by the occurrence of DVT [1,2,4,5,30,31,32,33,34].

**Figure 3:**
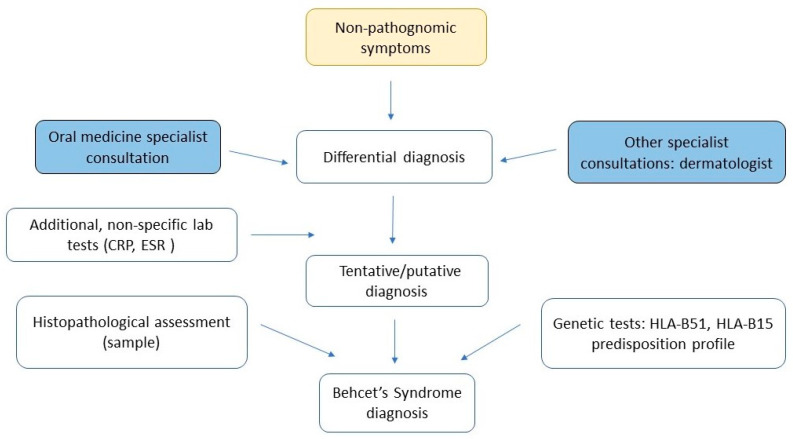
The proposed, simplified diagnostic process in BD detection, based on mucocutaneous manifestations.

Although there are no specific point-of-care diagnostics for BD, plausibly, the clinical, non-characteristic findings support primarily the diagnostic process (Figure 3) based on diagnostic criteria, and persistently high CRP and ESR levels, as identified in our case study [42]. Parsaei et al. observed that both CRP and ESR can be closely related to an active state of BD predicting active vascular manifestations of BD exacerbation [43]. Increased oxidative protein products have been suggested as a new BD activity biomarker by Yezici et al. [44]. It must be stressed that clinical examination, including intraoral assessment, and correlation of findings (e.g., oral/genital ulcerations) with patient’s medical history are sufficient to diagnose BD. However, in some cases, additional tests, such as lesion biopsy and histopathological investigation may be performed but not required during a standard diagnostic process, to rule out other conditions.

The histopathological assessment of oral lesions performed in this case contributed to the diagnostic process and understanding of BD, offering valuable insights into the underlying inflammatory processes, and aided in differential diagnosis. By describing the histological features observed, such as vasculitis, perivascular inflammatory infiltrates, it provided clinicians with important diagnostic information. It helped to guide diagnostic decisions, ruling out other potential conditions with similar clinical presentations. Overall, this case report highlights the clinical usefulness of histopathological assessment in the management of Behçet’s disease, emphasizing its adjunct role in diagnosis.

Understanding the genetic susceptibility to various rare diseases can help pinpoint the cause of existing, underdiagnosed disorders which would lead to exact diagnoses and superior outcomes. The utilisation of advanced HLA genotyping using testing for gene expression provides additional support due to the evidence-based association with genetic markers, more specifically, the presence of the HLA-B51 allele, especially in some ethnic groups, such as the Turks [45]. The main described genetic profile related to BD was HLA-B51 [1,5,12,13,46,47], present in about 60% of persons affected by BD. In our case study, although the HLA-B51 expression was not observed, the patient was positive for the HLA-B15 allele, which is associated with the late occurrence of BD (after 30 years of age), particularly in men [48]. In line with this notion, genome-wide association studies recently revealed the contribution of other genes related to the immune system, such as: IL10, IL23R, KLRC4, ERAP1, STAT4, CCR1-CCR3, and TNFAIP3 [47]. The results of the study conducted by Burillo-Sanz et al. [49], assessing functional polymorphism in genes associated with autoinflammatory diseases (CECR1, MEFV, MVK, NLRP3, NOD2, and PSTPIP1), suggest that a substantial fraction of patients with BD have rare variants of these genes, with a suspected link of MEFV with BD modulated by HLA molecules. The review of studies concerning the usefulness of genetic profiling in BD is presented in Table 3.

Since BD has no pathognomonic clinical characteristics, and because oral symptoms are common in other oral conditions, particularly immunologically mediated diseases, such as pemphigus, mucous membrane pemphigoid, and Stevens–Johnson syndrome [4,5,28,29], collaborative efforts among various specialists, including dermatologists, ophthalmologists, rheumatologists, neurologists, and gastroenterologists are important in evaluating patients with suspected BD. The diagnostic process should also consider a wide range of systemic diseases that may present with similar manifestations, including microscopic polyangiitis, polyarteritis nodosa, inflammatory bowel disease, celiac disease, systemic lupus erythematous, herpes simplex virus infection, reactive arthritis, Sjogren syndrome, immunobullous skin diseases, multiple sclerosis, and sarcoidosis [1,5,38]. Immunofluorescence and immunohistochemical methods can be helpful in supporting the differential diagnosis of mucocutaneous lesions (papulopustular eruptions, recurrent oral aphthous ulcerations) [29].

In the diagnostic process, the presence of characteristic clinical features, including intraoral findings, are essential and they have been obtained through the set of oral manifestations as presented in our report. Oral health status has also been linked to the severity of BD in several studies [57,58,59]. Using a structured diagnostic pathway, two main diagnostic recommendations are used for BD, including the ISG [36] and ICBD [37]. Indeed, in our presented case, the diagnostic confirmation was based on both criteria, since the patient had a history of recurrent oral and genital ulcers, in addition to the occurrence of skin lesions (pseudofolliculitis), thus fulfilling the ISG criteria (Table 2). However, it is important to note that the ISG criteria may lack specificity and have long intervals between the manifestation of mucocutaneous lesions, while remarkably similar signs and symptoms can be observed in a variety of other diseases. Despite the increased sensitivity of the ICBD, it may result in over-diagnosis if solely relied upon, especially when scoring predominantly based on oral and genital ulcerations (scoring > 4, Table 4). Numerous studies also reported the loss of specificity compared to the ISG criteria.

Overall, regarding the ICBD criteria, the patient presented in our case report obtained a score of six points, based on the criteria that the occurrence of oral and genital ulcerations confers four points and the presence of skin lesions (pseudofolliculitis) and vascular events (thrombosis of the iliac vein and inferior vena cava) each confer one point. As a result, the patient fitted the highest level of diagnostic verification of BD according to the scale adopted by the ICBD guidelines.

Although a wide range of signs and symptoms may be present during the assessment process, intraoral non-specific ulcers play a predominant role and are considered key features according to commonly used diagnostic recommendations [36,37]. Reportedly, oral lesions represent the initial symptoms of BD in 47% to 86% of cases, usually preceding other local or systemic manifestations. Furthermore, they may persist for a longer period, even when the most severe manifestations of the disease are in remission. Thus, oral manifestations can be used for clinical monitoring as a marker of disease activity and efficacy of the treatment implemented [57,59]. The oral ulcers associated with BD are typically painful, round, smaller than 10 mm in diameter, shallow or deep, with yellowish fibrinopurulent background, with an evident erythematous halo [28,42]. They can develop spontaneously or after minor trauma (positive pathergy test), dental treatment, or both, causing an immune response against oral microbiota microorganisms, especially Streptococcus sanguinis. Oral lesions usually manifest as a trigger for systemic manifestations in genetically susceptible individuals [45,46,47,48].

Similarly, the presence of multiple ulcers dispersed throughout the oral cavity, along with a history of genital ulcers, an evident erythematous halo and background with yellowish-white pseudomembrane, pseudofolliculitis, and DVT supported the diagnosis of the described case. Histopathological examination of the biopsy from the buccal mucosa lesion revealed an intense mixed inflammatory infiltrate, congested vessels, and areas of vasculitis in the underlying connective tissue (ulcerated epithelium) and were essential elements for the diagnosis. These findings align with previous studies on the microscopic features of BD. According to Emmi et al., BD vasculitis is characterised by the presence of an inflammatory infiltrate composed of neutrophils and lymphocytes, arising in a perivascular manner. This pattern was found mainly in the mucous and ocular lesions of the disease which corroborates our findings [60].

As there is no specific therapy for BD, treatment focuses on managing signs and symptoms through immunosuppressive pharmacotherapy [1,4,5,6,61]. Genital and oral ulcers are typically treated with topical corticosteroids, while systemic corticosteroids can be required during exacerbation of mucocutaneous lesions [28]. Colchicine can be employed to prevent relapses, especially when the erythema nodosum or genital ulcers are the primary lesions. In cases involving DVT, treatment with systemic corticosteroids and immunosuppressants is used as they have shown lower recurrent rate compared to anticoagulants. The use of certain types of anticoagulants for DVT in BD remains controversial [61,62].

In the specific case discussed, the patient was treated with continuous daily systemic prednisolone and colchicine along with weekly administration of methotrexate. This treatment approach was chosen due to the patient’s active oral lesions, history of recurrent genital lesions, and DVT. The treatment strategy resulted in complete remission of oral lesions, and the absence of recurrence of any other manifestations over eight months leading to an improvement in the patient’s quality of life. Tumour necrosis factor (TNF) inhibiting/blocking agents, such as adalimumab and infliximab have shown clinical success in treating BD patients [6] (Table 5). Recognising the various manifestations of BD has contributed to the successful pharmacotherapy in managing the disease. Understanding of the distinct clinical features of BD and the pathogenesis of the hypercoagulable/prothrombotic state associated with oral manifestations may lead to the development of effective novel therapeutic methods. Overall, the early detection of potential life-threatening complications and multidisciplinary management are crucial in addressing the diagnostic challenges and the impact on quality of life associated with BD.

Inherent to the nature of case reports, there are certain limitations that should be acknowledged. The generalizability of findings from a single case is inherently limited, as individual patient characteristics can vary significantly. Therefore, caution should be exercised when applying the findings of a single case report to broader patient populations. Additionally, selection bias may exist in case reports, as they often highlight unique or atypical cases that may not be representative of the overall patient population. Arguably, case reports are prone to publication bias, where cases with more interesting or positive outcomes are more likely to be published, potentially skewing the overall understanding of a particular condition or treatment approach. Despite these limitations, case reports play a valuable role in providing initial observations, generating hypotheses, and offering insights into rare or unusual clinical presentations, ultimately contributing to the collective knowledge base in healthcare.

### Implications and Future Perspectives

This case emphasizes an optimal, and patient-centred diagnostic approach for BD, involving different specialties supported by regional centres with oral medicine teams. This would enable prompt referral and redirection to secondary services to commence appropriate care, thereby, preventing severe systemic health consequences that can be potentially life-threatening. Despite the existing, well recognized international clinical criteria for BD, this condition has been recently described as a ‘great masquerader’ since it may have variable presentations and can be frequently misdiagnosed [81]. This report highlights the importance of meticulous clinical dental and medical examination to facilitate definite diagnosis and the crucial role of dental practitioners in the early detection of most common BD manifestations, based on intraoral findings. The implementation of artificial intelligence integrated into the network of healthcare services can assist in recognising, matching and flagging certain symptoms and known characteristics which could improve diagnostic accuracy in patients suffering from oral conditions. The authors call for future genetics-based approaches to enhance diagnostic yield and elucidate the molecular mechanisms of the disease. Modern techniques such as short-read RNA genome sequencing, metabolomics, proteomics and methyl profiling are suggested to trigger a paradigm shift in diagnostic precision for especially underrepresented populations [82,83].

Nevertheless, this modern approach to medical management can also be applied to the precise assessment and verification of histological samples (if justified for a diagnostic process in a complex cases) as an adjunct modality in the laboratory domain. Undoubtedly, in addition to aiding diagnosis, the passage suggests that the investment in advanced target-specific immunohistopathological and genetics testing, including gene-expression based methods can be employed for more precise assessment. These methods could play a pivotal role in timely and precise diagnoses of less common disorders, especially those with an autoimmune origin. The efforts of the scientific community must be intensified to support the BD diagnosis based on future research collaboration, multi-centre, multi-ethnic projects, and laboratory studies to enhance point-of-care testing. While clinical appearance of BD is frequently masquerading as other oral or systemic entities, clinicians need to be vigilant and appropriately trained in comprehensive differential diagnosis. This case report warrants larger population-based studies to improve the diagnostic protocol for heterogeneous disorders with non-characteristic manifestations and genetic predisposition.

## 4. Conclusions

The key role of dental professionals to support a diagnosis of systemic diseases seems better recognised nowadays, with the aim of achieving the best outcome for patients. Oral manifestations associated with underlying systemic, immune-driven pathologies have a significant contribution to the BD diagnostic pathway. Similar to the severe systemic manifestations of BD, localized oral ulcers can be debilitating, causing significant impairment in daily activities and worsening nutritional status, resulting in the deterioration of quality-of-life. This study underscores the essential contribution of oral medicine specialists, and a multidisciplinary approach to the diagnosis and management of systemic diseases like BD. It emphasizes the significance of oral manifestations in the diagnostic pathway and highlights the impact of BD on patients’ quality of life, as well as the importance of close cooperation of allied health professionals in BD management. The interdisciplinary approach was a fundamental aspect, considering that during differential diagnosis stages, the contribution of professionals with different specialties was pivotal to provide adequate multidisciplinary support. Advanced diagnostic methods are urgently required to facilitate prompt and accurate diagnoses of systemic inflammatory conditions with predominant oral manifestations, affecting various health aspects.

## Figures and Tables

**Table 2:** International Study Group diagnostic criteria of Behçet’s disease (1990).

Mucocutaneous Lesion
Recurrent oral ulceration, at least three episodes over the past 12 months, plus two of the following ‘hallmark’ symptoms:
Recurrent genital ulceration observed by patient or physician Eye inflammation/lesions observed by ophthalmologist Cutaneous lesion observed by physician or post-adolescent patients not receiving corticosteroids treatment Positive pathergy reaction test read by physician at 24 to 48 h

**Table 3:** The review of genetic characteristics supporting BD diagnosis.

Authors/Year	Study Design	Conclusions
Ohno et al., 1982 [50]	Case–control	The study showed that Behçet’s disease is closely associated with HLA-Bw51. Relative risk for HLA-Bw51 was 6.0, and an individual with HLA-Bw51 is considered at greater risk of having Behçet’s disease develop: the risk being six times more than an individual without it.
Baricordi et al., 1986 [51]	Case–control	A significant increase in HLA-B51 (*p* less than 0.00001) and DRw52 (*p* = 0.045) with no significant difference between complete and incomplete syndrome was found. The involvement of B51 antigen as the main immunogenetic factor in the disease is suggested by the high value of relative risk (RR = 16.03)
Chang et al., 2001 [52]	Case–control	The prevalence of HLA-B51 in patients with BD was 55.7%, 16.1% in patients with recurrent aphthous stomatitis (RAS), and 15.7% in healthy controls. Compared to patients with RAS or healthy controls, prevalence of HLA-B51 in Korean patients with BD was much higher.
Choukri et al., 2001 [53]	Case–control	The predisposing effect of the B*51 was confirmed (30.2% in patients and 15.3% in controls, OR = 2.39, 95% CI (1.2–4.8), *p* = 0.015). Moroccan BD group also presented a previously unknown association with HLA-B*15 (25.6% of patients versus 11.7% of controls, OR= 2.59 (1.2–5.5), *p* = 0.05.
Pirim et al., 2004 [54]	Case–control	The result shows that the HLA-B51 frequency was significantly higher (58.66%) in the patient group, compared to that of the control group (18.51%) (OR = 6.245).
Mizuki et al., 2007 [55]	Case–control	HLA-B*51 was also significantly more frequent in BD patients (81.8%) than in controls (29.2%) (*p* = 0.0000007). Our results indicate that the major susceptibility gene for BD is HLA-B*51.
Menthon et al., 2009 [56]	Systematic review and meta-analysis	A total of 4800 patients with BD and 16,289 controls from 78 independent studies (published 1975–2007) were selected. The pooled OR of HLA-B51/B5 allele carriers to develop BD compared with noncarriers was 5.78 (95% CI 5.00–6.67). The strength of the association between BD and HLA-B51/B5, and its consistency across populations of various ethnicities, lends further support to this allele being a primary and causal risk determinant for BD.

**Table 4:** International Criteria for Behçet’s Disease (2013), modified and adapted (scoring > 4 indicates Behçet’s disease diagnosis).

Sign/Symptom	Points
Ocular lesions	2
Genital apthosis	2
Oral apthosis	2
Skin lesions	1
Neurological manifestations	1
Vascular manifestations	1
Positive pathergy test	1 *

* Primary ISG scoring does not include pathergy test. An additional one point is assigned for positive pathergy test if tested.

**Table 5:** The results of selected studies investigating the effect of therapeutic agents in treatment of BD (oral ulcers and systemic).

Oral Ulcers Treatment	Dose/Duration	Results	Authors/Year Study Design
Interferon alpha vs. Placebo	1 × 10^5^ U/g three times a day ∥ 24 weeks	No difference between the groups	Hamuryudan et al., 1991–RCT [63]
Cyclosporine vs. Placebo	70 mg per g of orobase ∥ 8 weeks	No difference between the groups	Ergun et al., 1997–RCT [64]
Sucralfate vs. Placebo	4 times a day ∥ 3 months	Decreases frequency, healing time and pain of oral ulcers	Alpsoy et al., 1999–RCT [65]
Triamcinolone acetonide vs. Phenytoin	3 times a day ∥ 1 week	Triamcinolone was more effective than phenytoin	Fani et al., 2012–RCT [66]
Pentoxifylline + Colchicine vs. Colchicine	1000 mg/d (4 doses) ∥ 14 days	Decrease in duration and pain of oral ulcers in pentoxifylline group	Hatemi et al., 2019–RCT [67]
**Systemic Treatment**	**Dose/Duration**	**Results**	**Authors/Year** **Study Design**
Colchicine vs. Placebo	1 mg/d ∥ 9 months 1–2 mg/d ∥ 2 years 1 mg/d ∥ 4 months	Decreases frequency of erythema nodosum, and effective on arthralgia Reduces the occurrence of genital ulcers, erythema nodosum and arthritis in women, and the occurrence of arthritis in men Improvement of oral ulcers, genital ulcers, erythema nodosum and papulopustular lesions in both genders	Aktulga et al., 1980–RCT [68] Yurdakul et al., 2001–RCT [69] Davatchi et al., 2009–RCT [70]
Cyclosporine vs. conventional treatments (prednisolone, chlorambucil)	5–10 mg/kg/d ∥ 3 years	Cyclosporine more effective than conventional therapy in ocular disease. Conventional treatments were superior to cyclosporine for the control of skin lesions and arthritis	BenEzra et al., 1988–RCT [71]
Cyclosporine vs. Colchicine	10 mg/kg/d + 1 mg/d ∥ 4 months	Cyclosporin more effective in treating ocular manifestations, oral ulcers, dermal lesions, and genital ulcers.	Masuda et al., 1989–RCT [72]
Methotrexate	7.5–12.5 mg/week ∥ 12 months Initial dose of 5.0–7.5 mg/week, increased by 2.5 mg every 2 weeks up to 5.0–15 mg/week ∥ 4 year	Beneficial effect in the treatment of progressive Neuro-Behçet Prevent the progression of the neuropsychiatric manifestations of Neuro-Behçet by markedly decreasing CSF IL-6 levels	Hirohata et al., 1988-Open-label study [73] Kikuchi et al., 2003-Open-label study [74]
Thalidomide vs. Placebo	300 mg/day ∥ 4 weeks	Effective on oral ulcers, genital ulcers and follicular lesions.	Hamuryudan et al., 1998–RCT [75]
Etanercept vs. Placebo	25 mg twice a week ∥ 4 weeks	Mean numbers of oral ulcers, nodular lesions, and papulopustular lesions were less in the etanercept group compared to the placebo group at all weekly evaluations, except for the second week for papulopustular lesions	Melikoglu et al., 2005–RCT [76]
Corticosteroids vs. Placebo	40 mg/every 3 weeks ∥ 27 weeks	No beneficial effect on genital ulcers. Useful in controlling erythema nodosum lesions, especially among the females	Mat et al., 2006–RCT [77]
Isotretinoin vs. Placebo	20 mg/d ∥ 12 weeks	Improvement in the clinical manifestations index, oral ulcers and skin manifestations parameters	Sharquie et al., 2013–RCT [78]
Interferon-α2b vs. glucocorticoids and immunosuppressives Adalimumab	0.3 μg/kg/w ∥ 26 weeks 40 mg subcutaneously, once every 2 weeks	Reduction in corticosteroid dose at 1-year, improved quality of life and trend to reduce immunosuppressive agents, with the addition of peginterferon-α-2b to the drug regime in patients with BS with ocular and systemic involvement Adalimumab is very a very effective and safe option for treatment of patients with severe and resistant Behçet’s uveitis, providing an appropriate and long-term control of ocular inflammation.	Lightman et al., 2015–RCT [79] Interlandi et al., 2014–Retrospective follow-up study [80]

## Data Availability

The original clinical information (anonymized) available on request.

## References

[1] Adil A., Goyal A., Quint J.M. (2022). Behcet Disease. StatPearls [Internet].

[2] Nair J.R., Moots R.J. (2017). Behcet’s disease. Clin. Med..

[3] Behcet H. (1937). Über rezidivierende, aphthöse, durch ein virus verursachte Geschwüre am Mund, am Auge und an den Genitalien. Dermatol. Wochenschr..

[4] Ali S., Nagieb C.S., Fayed H.L. (2022). Effect of Behcet’s disease-associated oral ulcers on oral health related quality of life. Spec. Care. Dentist..

[5] Davatchi F., Chams-Davatchi C., Shams H., Shahram F., Nadji A., Akhlaghi M., Faezi T., Ghodsi Z., Sadeghi Abdollahi B., Ashofteh F. (2017). Behcet’s disease: Epidemiology, clinical manifestations, and diagnosis. Expert Rev. Clin. Immunol..

[6] Greco A., De Virgilio A., Ralli M., Ciofalo A., Mancini P., Attanasio G., de Vincentiis M., Lambiase A. (2018). Behçet’s disease: New insights into pathophysiology, clinical features and treatment options. Autoimmun. Rev..

[7] McGonagle D., Aydin S.Z., Gül A., Mahr A., Direskeneli H. (2015). ‘MHC-I-opathy’—Unified concept for spondyloarthritis and Behçet disease. Nat. Rev. Rheumatol..

[8] Giza M., Koftori D., Chen L., Bowness P. (2018). Is Behçet’s disease a ‘class 1-opathy’? The role of HLA-B*51 in the pathogenesis of Behçet’s disease. Clin. Exp. Immunol..

[9] Pineton de Chambrun M., Wechsler B., Geri G., Cacoub P., Saadoun D. (2012). New insights into the pathogenesis of Behçet’s disease. Autoimmun. Rev..

[10] Kapsimali V.D., Kanakis M.A., Vaiopoulos G.A., Kaklamanis P.G. (2010). Etiopathogenesis of Behçet’s disease with emphasis on the role of immunological abberations. Clin. Rheumatol..

[11] Rodríguez-Carrio J., Nucera V., Masala I.F., Atzeni F. (2021). Behçet disease: From pathogenesis to novel therapeutic options. Pharmacol. Res..

[12] Hatemi G., Seyahi E., Fresko I., Talarico R., Uçar D., Hamuryudan V. (2022). Behçet’s syndrome: One year in review 2022. Clin. Exp. Rheumatol..

[13] Takeuchi M., Kastner D.L., Remmers E.F. (2015). The immunogenetics of Behçet’s disease: A comprehensive review. J. Autoimmun..

[14] Salmaninejad A., Zamani M.R., Shabgah A.G., Hosseini S., Mollaei F., Hosseini N., Sahebkar A. (2019). Behçet’s disease: An immunogenetic perspective. J. Cell Physiol..

[15] Wakefield D., Cunningham E.T., Tugal-Tutkun I., Khairallah M., Ohno S., Zierhut M. (2012). Controversies in Behçet disease. Ocul. Immunol. Inflamm..

[16] Ni Mhuircheartaigh O., Hunt C., Huang J., Kumar N., Matteson E.L. (2012). Diagnostic dilemma in CNS Behcet’s disease. BMJ Case Rep..

[17] Skef W., Hamilton M.J., Arayssi T. (2015). Gastrointestinal Behçet’s disease: A review. World J. Gastroenterol..

[18] Bernabé E., Marcenes W., Mather J., Phillips C., Fortune F. (2010). Impact of Behçet’s syndrome on health-related quality of life: Influence of the type and number of symptoms. Rheumatology.

[19] Hatemi G., Seyahi E., Fresko I., Talarico R., Hamuryudan V. (2020). One year in review 2020: Behçet’s syndrome. Clin. Exp. Rheumatol..

[20] Kiraz S., Ertenli I., Oztürk M.A., Haznedaroğlu I.C., Celik I., Calgüneri M. (2002). Pathological haemostasis and “prothrombotic state” in Behçet’s disease. Thromb. Res..

[21] Butta N.V., Fernández-Bello I., López-Longo F.J., Jiménez-Yuste V. (2015). Endothelial Dysfunction and Altered Coagulation As Mediators of Thromboembolism in Behçet Disease. Semin. Thromb. Hemost..

[22] Yurdakul S., Hekim N., Soysal T., Fresko I., Bavunoglu I., Ozbakir F., Tabak F., Melikoglu M., Hamuryudan V., Yazici H. (2005). Fibrinolytic activity and d-dimer levels in Behçet’s syndrome. Clin. Exp. Rheumatol..

[23] Demirer S., Sengül N., Yerdel M.A., Tüzüner A., Ulus A.T., Gürler A., Bergqvist D., Siegbahn A., Karacagil S. (2000). Haemostasis in patients with Behçet’s disease. Eur. J. Vasc. Endovasc. Surg..

[24] Chambers J.C., Haskard D.O., Kooner J.S. (2001). Vascular endothelial function and oxidative stress mechanisms in patients with Behçet’s syndrome. J. Am. Coll. Cardiol..

[25] Ataş H., Canpolat F., Eskioglu F. (2018). Evaluation of Mean Platelet Volume in Patients with Behcet’s Disease as an Indicator of Vascular Thrombosis. Arch. Iran. Med..

[26] Fernández-Bello I., López-Longo F.J., Arias-Salgado E.G., Jiménez-Yuste V., Butta N.V. (2013). Behçet’s disease: New insight into the relationship between procoagulant state, endothelial activation/damage and disease activity. Orphanet J. Rare Dis..

[27] Saadoun D., Wechsler B., Desseaux K., Le Thi Huong D., Amoura Z., Resche-Rigon M., Cacoub P. (2010). Mortality in Behçet’s disease. Arthritis Rheum..

[28] Marinho K.C.T., Giovani E.M. (2016). The treatment of oral lesions in Behçet’s Syndrome: Case report. Rev. Esp. Cirug. Oral Maxilofac..

[29] Gündüz O. (2012). Histopathological Evaluation of Behçet’s Disease and Identification of New Skin Lesions. Pathol. Res. Int..

[30] Calamia K.T., Wilson F.C., Icen M., Crowson C.S., Gabriel S.E., Kremers H.M. (2009). Epidemiology and clinical characteristics of Behçet’s disease in the US: A population-based study. Arthritis Rheum..

[31] Thomas T., Chandan J.S., Subramanian A., Gokhale K., Gkoutos G., Harper L., Buckley C., Chandratre P., Raza K., Situnayake D. (2020). Epidemiology, morbidity and mortality in Behçet’s disease: A cohort study using The Health Improvement Network (THIN). Rheumatology.

[32] Mohammad A., Mandl T., Sturfelt G., Segelmark M. (2013). Incidence, prevalence and clinical characteristics of Behcet’s disease in southern Sweden. Rheumatology.

[33] Sachetto Z., Mahayri N., Ferraz R.H., Costallat L.T.L., Bertolo M.B. (2012). Behçet’s disease in Brazilian patients: Demographic and clinical features. Rheumatol. Int..

[34] Savey L., Resche-Rigon M., Wechsler B., Comarmond C., Piette J.C., Cacoub P., Saadoun D. (2014). Ethnicity and association with disease manifestations and mortality in Behçet’s disease. Orphanet J. Rare Dis..

[35] Saleh Z., Arayssi T. (2014). Update on the therapy of Behçet disease. Ther. Adv. Chronic Dis..

[36] Criteria for Diagnosis of Behçet’s Disease (1990). International Study Group for Behçet’s Disease. Lancet.

[37] Blake T., Pickup L., Carruthers D., Damato E.M., Denniston A., Hamburger J., Maxton C., Mitton D., Murray P.I., Nightingale P. (2017). Birmingham Behçet’s service: Classification of disease and application of the 2014 International Criteria for Behçet’s Disease (ICBD) to a UK cohort. BMC Musculoskelet. Disord..

[38] Behçet’s UK How Is Behçet’s Diagnosed?. https://behcetsuk.org/how-is-behcets-diagnosed/.

[39] International Team for the Revision of the International Criteria for Behçet’s Disease (ITR-ICBD) (2014). The International Criteria for Behçet’s Disease (ICBD): A collaborative study of 27 countries on the sensitivity and specificity of the new criteria. J. Eur. Acad. Dermatol. Venereol..

[40] Farahangiz S., Sarhadi S., Safari A., Borhani-Haghighi A. (2012). Magnetic resonance imaging findings and outcome of neuro-Behçet’s disease: The predictive factors. Int. J. Rheum. Dis..

[41] Davatchi F. (2012). Diagnosis/Classification Criteria for Behcet’s Disease. Pathol. Res. Int..

[42] Mumcu G., Karacayli Ü., Yay M., Aksoy A., Taş M.N., Armağan B., Sari A., Bozca B.C., Tekgöz E., Karadağ D.T. (2019). Oral ulcer activity assessment with the composite index according to different treatment modalities in Behçet’s syndrome: A multicentre study. Clin. Exp. Rheumatol..

[43] Parsaei A., Moradi S., Masoumi M., Davatchi F., Najafi A., Kooshki A.M., Hajighadery A., Akhlaghi M., Faezi T., Kavosi H. (2022). Predictive value of erythrocyte sedimentation rate and C-reactive protein in Behcet’s disease activity and manifestations: A cross-sectional study. BMC Rheumatol..

[44] Yazici C., Köse K., Caliş M., DemIr M., Kirnap M., Ateş F. (2004). Increased advanced oxidation protein products in Behçet’s disease: A new activity marker?. Br. J. Dermatol..

[45] Pekiner F.N., Aytugar E., Demirel G.Y., Oǧuz Borahan M. (2013). HLA-A, B (class I) and HLA-DR, DQ (class II) antigens in Turkish patients with recurrent aphthous ulceration and Behçet’s disease. Med. Princ. Pract..

[46] Yazici H., Chamberlain M.A., Schreuder I., D’Amaro J., Muftuoglu M. (1980). HLA antigens in Behçet’s disease: A reappraisal by a comparative study of Turkish and British patients. Ann. Rheum. Dis..

[47] Kirino Y., Bertsias G., Ishigatsubo Y., Mizuki N., Tugal-Tutkun I., Seyahi E., Ozyazgan Y., Sacli F.S., Erer B., Inoko H. (2013). Genome-wide association analysis identifies new susceptibility loci for Behçet’s disease and epistasis between HLA-B*51 and ERAP1. Nat. Genet..

[48] Gül A., Uyar F.A., Inanc M., Ocal L., Tugal-Tutkun I., Aral O., Koniçe M., Saruhan-Direskeneli G. (2001). Lack of association of HLA-B*51 with a severe disease course in Behçet’s disease. Rheumatology.

[49] Burillo-Sanz S., Montes-Cano M.-A., García-Lozano J.-R., Olivas-Martínez I., Ortego-Centeno N., García-Hernández F.-J., Espinosa G., Graña-Gil G., Sánchez-Bursón J., Juliá M.R. (2019). Behçet’s disease and genetic interactions between HLA-B*51 and variants in genes of autoinflammatory syndromes. Sci. Rep..

[50] Ohno S., Ohguchim M., Hirose S., Matsuda H., Wakisaka A., Aizawa M. (1982). Close association of HLA-Bw51 with Behçet’s disease. Arch Ophthalmol..

[51] Baricordi O.R., Sensi A., Pivetti-Pezzi P., Perrone S., Balboni A., Catarinelli G., Filippi F., Melchiorri L., Moncada A., Mattiuz P.L. (1986). Behcet’s disease associated with HLA-B51 and DRw52 antigens in Italians. Hum. Immunol..

[52] Chang H.K., Kim J.U., Cheon K.S., Chung H.R., Lee K.W., Lee I.H. (2001). HLAB51 and its allelic types in association with Behçet’s disease and recurrent aphthous stomatitis in Korea. Clin. Exp. Rheumatol..

[53] Choukri F., Chakib A., Himmich H., Hüe S., Caillat-Zucman S. (2001). HLAB*51 and B*15 alleles confer predisposition to Behçet’s disease in Moroccan patients. Hum. Immunol..

[54] Pirim I., Atasoy M., Ikbal M., Erdem T., Aliagaoglu C. (2004). HLA class I and class II genotyping in patients with Behcet’s disease: A regional study of eastern part of Turkey. Tissue Antigens.

[55] Mizuki N., Meguro A., Tohnai I., Gül A., Ohno S., Mizuki N. (2007). Association of major histocompatibility complex class I chain-related gene A and HLA-B alleles with Behçet’s disease in Turkey. Jpn. J. Ophthalmol..

[56] de Menthon M., Lavalley M.P., Maldini C., Guillevin L., Mahr A. (2009). HLA-B51/B5 and the risk of Behçet’s disease: A systematic review and meta-analysis of case-control genetic association studies. Arthritis Rheum..

[57] Mumcu G., Ergun T., Inanc N., Fresko I., Atalay T., Hayran O., Direskeneli H. (2004). Oral health is impaired in Behçet’s disease and is associated with disease severity. Rheumatology.

[58] Karacayli U., Mumcu G., Simsek I., Pay S., Köse O., Erdem H., Direskeneli H., Gunaydin Y., Dinc A. (2009). The close association between dental and periodontal treatments and oral ulcer course in Behcet’s disease: A prospective clinical study. J. Oral Pathol. Med..

[59] Yay M., Çelik Z., Aksoy A., Alibaz-Öner F., Inanç N., Ergun T., Direskeneli H., Mumcu G. (2019). Oral health is a mediator for disease severity in patients with Behçet’s disease: A multiple mediation analysis study. J. Oral Rehabil..

[60] Emmi G., Bettiol A., Silvestri E., Di Scala G., Becatti M., Fiorillo C., Prisco D. (2019). Vascular Behçet’s syndrome: An update. Intern. Emerg. Med..

[61] Alpsoy E., Leccese P., Emmi G., Ohno S. (2021). Treatment of Behçet’s Disease: An Algorithmic Multidisciplinary Approach. Front. Med..

[62] Ozguler Y., Leccese P., Christensen R., Esatoglu S.N., Bang D., Bodaghi B., Çelik A.F., Fortune F., Gaudric J., Gul A. (2018). Management of major organ involvement of Behçet’s syndrome: A systematic review for update of the EULAR recommendations. Rheumatology.

[63] Hamuryudan V., Yurdakul S., Rosenkaimer F., Yazici H. (1991). Inefficacy of topical alpha interferon in the treatment of oral ulcers of Behçet’s syndrome: A randomized, double blind trial. Br. J. Rheumatol..

[64] Ergun T., Gürbüz O., Yurdakul S., Hamuryudan V., Bekiroglu N., Yazici H. (1997). Topical cyclosporine-A for treatment of oral ulcers of Behçet’s syndrome. Int. J. Dermatol..

[65] Alpsoy E., Er H., Durusoy C., Yilmaz E. (1999). The use of sucralfate suspension in the treatment of oral and genital ulcerations of Behçet’s disease: A randomised, placebo-controlled and double-blind study. Arch. Dermatol..

[66] Fani M.M., Ebrahimi H., Pourshahidi S., Aflaki E., Shafiee Sarvestani S. (2012). Comparing the effect of phenytoin syrup and triamcinolone acetonide ointment on aphthous ulcers in patients with Behcet’s syndrome. Iran. Red. Crescent Med. J..

[67] Hatemi G., Yurttas B., Kutlubay Z., Cote T., Derkunt S.B., Yazici Y., Yazici H. (2019). Pentoxifylline Gel for Oral Ulcers in Patients with Behçet’s Syndrome. Arthritis Rheumatol..

[68] Aktulga E., Altaç M., Müftüoglu A., Ozyazgan Y., Pazarli H., Tüzün Y., Yalçin B., Yazici H., Yurdakul S. (1980). A double blind study of colchicine in Behcet’s disease. Haematologica.

[69] Yurdakul S., Mat C., Tüzün Y., Özyazgan Y., Hamuryudan V., Uysal Ö., Şenocak M., Yazici H. (2001). A double-blind trial of colchicine in Behcet’s syndrome. Arthritis Rheum..

[70] Davatchi F., Sadeghi Abdollahi B., Tehrani Banihashemi A., Shahram F., Nadji A., Shams H., Chams-Davatchi C. (2009). Colchicine versus placebo in Behçet’s disease: Randomized, double-blind, controlled crossover trial. Mod. Rheumatol..

[71] Benezra D., Cohen E., Chajek T., Friedman G., Pizanti S., De Courten C., Harris W. (1988). Evaluation of conventional therapy versus cyclosporine A in Behçet’s syndrome. Transplant. Proc..

[72] Masuda K., Nakajima A., Urayama A., Nakae K., Kogure M., Inaba G. (1989). Double-masked trial of cyclosporin versus colchicine and long-term open study of cyclosporin in Behcet’s disease. Lancet.

[73] Hirohata S., Suda H., Hashimoto T. (1998). Low-dose weekly methotrexate for progressive neuropsychiatric manifestations in Behcet’s disease. J. Neurol. Sci..

[74] Kikuchi K., Aramaki H., Hirohata S. (2003). Low dose MTX for progressive neuro-Behcet’s disease. A follow-up study for 4 years. Adv. Exp. Med. Biol..

[75] Hamuryudan V., Mat C., Saip S., Ozyazgan Y., Siva A., Yurdakul S., Zwingenberger K., Yazici H. (1998). Thalidomide in the treatment of the mucocutaneous lesions of the Behçet syndrome. A randomized, double-blind, placebo-controlled trial. Ann. Intern. Med..

[76] Melikoglu M., Fresko I., Mat C., Ozyazgan Y., Gogus F., Yurdakul S., Hamuryudan V., Yazici H. (2005). Short-term trial of etanercept in Behcet’s disease: A double blind, placebo controlled study. J. Rheumatol..

[77] Mat C., Yurdakul S., Ozyazgan Y., Uysal S., Uysal O., Yazici H. (2006). A double-blind trial of depot corticosteroids in Behçet’s syndrome. Rheumatology.

[78] Sharquie K.E., Helmi R.M., Noiami A.A., Al-Hayani R.K., Kadhom M.A. (2013). The therapeutic role of isotretinoin in the management of Behçet’s disease: A single-blinded, controlled therapeutic study. J. Drugs Dermatol..

[79] Lightman S., Taylor S.R.J., Bunce C., Longhurst H., Lynn W., Moots R., Stanford M., Tomkins-Netzer O., Yang D., Calder V.L. (2015). Pegylated interferon-alpha-2b reduces corticosteroid requirement in patients with Behcet’s disease with upregulation of circulating regulatory T cells and reduction of Th17. Ann. Rheum. Dis..

[80] Interlandi E., Leccese P., Olivieri I., Latanza L. (2014). Adalimumab for treatment of severe Behçet’s uveitis: A retrospective long-term follow-up study. Clin. Exp. Rheumatol..

[81] Nordstrom E., Fischer M. (2014). The great masquerader: Behcet’s disease. BMJ Case Rep..

[82] Marwaha S., Knowles J.W., Ashley E.A. (2022). A guide for the diagnosis of rare and undiagnosed disease: Beyond the exome. Genome Med..

[83] Bettiol A., Emmi G., Liying L., Sofi F., Wallace G.R. (2023). Microbiome in Behcet’s syndrome. Clin. Immunol..

